# Modelling speech reception thresholds and their improvements due to spatial noise reduction algorithms in bimodal cochlear implant users

**DOI:** 10.1016/j.heares.2022.108507

**Published:** 2022-07

**Authors:** Ayham Zedan, Tim Jürgens, Ben Williges, David Hülsmeier, Birger Kollmeier

**Affiliations:** a Medizinische Physik and Cluster of Excellence “Hearing4all,” Carl-von-Ossietzky Universität Oldenburg, Oldenburg, Germany; b Institut für Akustik, Technische Hochschule Lübeck, Lübeck, Germany; c SOUND Lab & Cambridge Hearing Group, Department of Clinical Neurosciences, University of Cambridge, Cambridge, United Kingdom

**Keywords:** Cochlear implants, Speech recognition, Beamformers, Modelling, Bimodal

## Abstract

Spatial noise reduction algorithms (“beamformers”) can considerably improve speech reception thresholds (SRTs) for bimodal cochlear implant (CI) users. The goal of this study was to model SRTs and SRT-benefit due to beamformers for bimodal CI users. Two existing model approaches varying in computational complexity and binaural processing assumption were compared: (i) the framework of auditory discrimination experiments (FADE) and (ii) the binaural speech intelligibility model (BSIM), both with CI and aided hearing-impaired front-ends. The exact same acoustic scenarios, and open-access beamformers as in the comparison clinical study Zedan et al. (2021) were used to quantify goodness of prediction. FADE was capable of modeling SRTs ab-initio, i.e., no calibration of the model was necessary to achieve high correlations and low root-mean square errors (RMSE) to both, measured SRTs (r = 0.85, RMSE = 2.8 dB) and to measured SRT-benefits (r = 0.96). BSIM achieved somewhat poorer predictions to both, measured SRTs (r = 0.78, RMSE = 6.7 dB) and to measured SRT-benefits (r = 0.91) and needs to be calibrated for matching average SRTs in one condition. Greatest deviations in predictions of BSIM were observed in diffuse multi-talker babble noise, which were not found with FADE. SRT-benefit predictions of both models were similar to instrumental signal-to-noise ratio (iSNR) improvements due to the beamformers. This indicates that FADE is preferrable for modeling absolute SRTs. However, for prediction of SRT-benefit due to spatial noise reduction algorithms in bimodal CI users, the average iSNR is a much simpler approach with similar performance.

## Highlights

• This paper compares two modelling approaches to predict the speech recognition ability of bimodal CI users and the benefit of using beamformers.
• The modelling approaches vary in computational complexity and fitting requirements.
• A complex cafeteria spatial scenario with three localized single noise source scenario and a diffuse multi-talker babble noise is used.
• The automatic speech recognizer is more accurate across the different spatial scenarios and noise types and requires less fitting compared to the statistical modelling approach.

## 1 Introduction

The number of bimodal cochlear implant (CI) users, i.e., patients who use a hearing aid (HA) contralateral to their implanted ear, has substantially increased in the last 20 years (Leigh, Moran, Hollow, Dowell, 2016, Zaleski-King, Goupell, Barac-Cikoja, Bakke, 2019). Simultaneous usage of CI and HA has been shown to improve both, speech intelligibility (Ching, Incerti, Hill, van Wanrooy, 2006, Gifford, Dorman, Sheffield, Teece, Olund, 2014, Hoppe, Hocke, Digeser, 2018, Williges, Wesarg, Jung, Geven, Radeloff, Jürgens, 2019) and localization performance (Ching, Incerti, Hill, Hill, 2004, Dunn, Tyler, Witt, 2005) in comparison to usage of either device alone. Despite the benefit of bimodal hearing device usage, their overall speech-in-noise performance is still much poorer than the performance of normal-hearing (NH) listeners in the same acoustic scenarios (Weissgerber, Rader, Baumann, 2017, Williges, Wesarg, Jung, Geven, Radeloff, Jürgens, 2019). One reason for this poorer overall performance is that the combination of electrically stimulated and acoustic hearing across ears is much different from the binaural processing in NH listeners, for which some studies have found evidence: In a review of behavioural studies, van Hoesel (2012) found a complementary use of information across CI and HA for bimodal CI users due to the largely different cues available in both modalities. Whereas the HA supplies the user with low-frequency information with fine time-structure, the CI outputs mainly temporal envelope information and covers more the higher frequencies. Gifford et al. (2014) found absence of binaural squelch in bimodal users, and Francart et al. (2008) found much higher interaural time difference (ITD) thresholds in bimodal CI users than observed in NH listeners. Both results point towards the dysfunction or absence of a binaural processing stage acting on the fine time structure of the right and left signals, which usually provides binaural release of masking (Durlach, 1963) and sound localization ability (Jeffress, 1948, Kock, 1950) in NH listeners. With regard to spatial speech-in-noise performance, Williges et al. (2019) found mainly task-specific better-ear-listening within a group of bimodal CI users. The better performing ear is defined as the ear that would result in the best speech understanding outcome for the listener in a certain scenario or condition. For bimodal CI users, what is the better performing ear depends on the spatial scenario. Only with both poor and similar speech-in-noise performance across ears, significant benefits beyond better-ear-listening can be expected (Jürgens, Wesarg, Oetting, Jung, Williges, 2021, Yoon, Shin, Gho, Fu, 2015). The difference between NH and bimodal CI users in behavioural studies is further supported by Balkenhol et al. (2020), who found differences in cortical processing between bimodal CI users and NH listeners using objective methods.

Bimodal CI users experience a considerable benefit when using spatial noise reduction algorithms (‘beamformers’) as has been shown in multiple studies that tested their speech-in-noise performance in simplified and realistic acoustic scenarios (Buechner, Dyballa, Hehrmann, Fredelake, Lenarz, 2014, Devocht, Janssen, Chalupper, Stokroos, George, 2016, Ernst, Anton, Brendel, Battmer, 2019, Vroegop, Homans, Goedegebure, Dingemanse, Immerzeel, van der Schroeff, 2018, Weissgerber, Rader, Baumann, 2017, Zedan, Jürgens, Williges, Kollmeier, Wiebe, Galindo, Wesarg, 2021). The simplest group of beamformers are monaural beamformers (i.e., implemented in a single hearing device) which use the signals of two or more microphones to reduce the noise on one side of the listener, e.g., only the HA side or only CI side (Ernst et al., 2019). Binaural beamformers, on the other hand, utilize the signals of the microphones on both sides of the listener to provide binaural output ((Doclo et al., 2015). Owing to the larger distance across the microphones and the head shadow effect, they can outperform monaural beamformers (Baumgärtel, Hu, Krawczyk-Becker, Marquardt, Herzke, Coleman, Adiloglu, Bomke, Plotz, Gerkmann, Doclo, Kollmeier, Hohmann, Dietz, 2015, Bitzer, Simmer, Kammeyer, 1999). Most CI and HA devices have monaural beamformers, including the ones used by bimodal CI users. Studies comparing the two types of beamformers with bimodal CI users showed that binaural beamformers outperform monaural beamformers, especially in complex acoustic scenes by about 2–4 dB additional SRT-benefit (Ernst, Anton, Brendel, Battmer, 2019, Zedan, Jürgens, Williges, Kollmeier, Wiebe, Galindo, Wesarg, 2021).

Most of the aforementioned beamformer studies with bimodal CI users used commercially available CIs and HAs, which includes signal processing that is not open-access, but is held company-secret. This impedes the understanding about the interaction that spatial noise reduction algorithms and bimodal CI listening has, because both signal processing and physiologic mechanisms are unknown. An exception is the data from Zedan et al. (2021), who used open-access implementations of spatial noise processing algorithms within the Master Hearing Aid (MHA, Grimm et al. 2006). Furthermore, the recruited bimodal CI users all were implanted on the right side and used the same CI speech processor and signal processing strategy, reducing confounds from ear-of-entry and coding strategies. In addition, reproducible, albeit realistic acoustic scenes were taken in Zedan et al. (2021) by using virtual acoustics and providing the signals to the CI and the HA contralaterally via direct audio input. This way of presenting the signal to the participants provides better reproducibility and, in addition controls for their head movements, because unconscious head movements do not change the input signal-to-noise ratios (SNRs). Their study can be seen as belonging to a series of studies using the same approach and preprocessing algorithms in bilateral CI users (Baumgärtel et al., 2015a), bilateral HA users and NH listeners (Völker et al., 2015). Therefore, the experimental dataset with actual bimodal CI users by Zedan et al. (2021) provides a good basis to study the interaction between spatial noise algorithms and bimodal CI listening.

Speech intelligibility models are an integral part in the process of developing hearing loss treating technology (e.g., Byrne et al. 2001) and investigating the functional processes underlying it (Kollmeier and Kiessling, 2018). For speech intelligibility modeling, the dataset of Zedan et al. (2021), however, imposes particular challenges that have not been addressed by speech intelligibility models before, because (1) bimodal CI users perform a combination of electric and acoustic listening across ears and (2) adaptive beamforming in realistic rooms and subsequent compression algorithms within CI and HA result in a highly nonlinear signal processing. The present study therefore focuses on the comparison of two different model approaches that both have been very successful in their field, and that both need respective modifications to be able to address this dataset.

Standing in the tradition of microscopic, i.e., phoneme-recognition-based (Jürgens and Brand, 2009) and word-recognition-based (Holube and Kollmeier, 1996) speech intelligibility models, the framework for auditory discrimination experiments (FADE, Schädler et al. 2016b) was designed as a monaural model for psychoacoustic and speech intelligibility experiments using an automatic speech recognizer (ASR) backend. FADE successfully predicts speech-in-noise performance of NH listeners using both the widely-used German matrix sentence test, i.e., the Oldenburg sentence test (Schädler et al., 2015), as well as matrix sentence tests in different languages (Schädler et al., 2016a). Moreover, FADE predicts the effects of sensorineural hearing-impairment from mild to profound on speech-in-noise performance across a large range of levels and signal-to-noise ratios (SNRs) (Kollmeier et al., 2016). In combination with a physiologically plausible CI front-end feature extraction (Fredelake and Hohmann, 2012), FADE was able to predict the unilateral SRTs of CI users (Jürgens et al., 2018) and SRTs of monaural combinations of electric and acoustic listening that occur when CI users have retained acoustic hearing in the implanted ear (Zamaninezhad et al., 2017). FADE also successfully predicts the effects of noise reduction algorithms on SRTs of moderately hearing-impaired listeners on group averages (Schädler et al., 2018) and more recently also for individual hearing-impaired listeners (Hülsmeier, Buhl, Wardenga, Warzybok, Schädler, Kollmeier, 2021, Schädler, Hülsmeier, Warzybok, Kollmeier, 2020). However, being a monaural model, FADE has not yet been applied to model bimodal speech intelligibility, i.e., a combination of electric and acoustic listening across ears.

The binaural speech intelligibility model (BSIM, Beutelmann and Brand 2006), in contrast, was particularly designed to model binaural speech intelligibility for the application in different rooms. BSIM features an equalization-cancellation algorithm (Durlach, 1963) that effectively models binaural processing on the fine time structure of right and left signals, uncovered by binaural psychoacoustic experiments. BSIM successfully predicts the effect of different room acoustics on NH and HI listeners speech intelligibility in stationary noise (Beutelmann and Brand, 2006) and was extended as a short-term version BSIM2010 for modulated noises (Beutelmann et al., 2010) with empirically-supported binaural bandwidths, within which the actual binaural processing takes place (Beutelmann et al., 2009). BSIM was furthermore used to predict SRTs of vocoder experimental data simulating bimodal CI, bilateral CI and other combinations of simulated electric and acoustic hearing (Williges, Dietz, Hohmann, Jürgens, 2015, Zedan, Williges, Jürgens, 2018) and extended for strongly fluctuating noises (Hauth and Brand, 2018). An important characteristic of BSIM is that BSIM needs to be calibrated, i.e. fixed, to at least one experimental condition, which usually is the SRT with frontal co-located speech and noise for NH listeners. All predictions are then made relative to this calibration point. Another characteristic is that BSIM2010 needs to have access to the clean speech and noise signals a-priori to be able to calculate SNRs from which the actual SRT is inferred. Since dynamic range compression and beamformers are known to change the SNRs due to their inherent adaptive and nonlinear processing, BSIM so far was not applied to a dataset with such preprocessing algorithms on actual patients.

The goal of this study was to compare the two model approaches FADE and BSIM for the prediction of SRTs and SRT-benefit due to beamformers in bimodal CI users. Therefore, the exact same spatial conditions and details about signal processing in the devices were used in both of the model approaches as in the experimental comparison study of Zedan et al. (2021). The present study applied as-simple-as-possible modifications to the models to be able to preserve their proven features, but still realistically addressing the challenge that the dataset in Zedan et al. (2021) presents.

## 2 Methods

### 2.1 Experimental data

Zedan et al. (2021) reported on absolute SRTs, spatial release from masking (SRM), and SRT-improvements achieved by using a monaurally implemented beamformer (adaptive differential microphone, ADM) and a binaurally implemented beamformer (realized as a minimum variance distortionless response, MVDR, algorithm) of bimodal CI users. Virtual acoustics was used to realize four different spatial scenarios inside a realistic cafteria. They used the MHA (Grimm et al., 2006) to apply the beamformer algorithms and to simulate hearing aid processing. The so-processed signal was delivered to the CI speech processor Cochlear CP910 using an auxiliary input cable and to the contralateral ear using an in-ear headphone (Type: Etymotic ER-4). The SRTs measured in their study will be used as a direct comparison to the SRTs predicted by the model. The algorithms (both beamformers and hearing aid amplification and compression), spatial scenarios, and procedures used in the models of the present study are identical to the settings used in their study.

**Fig. 1:**
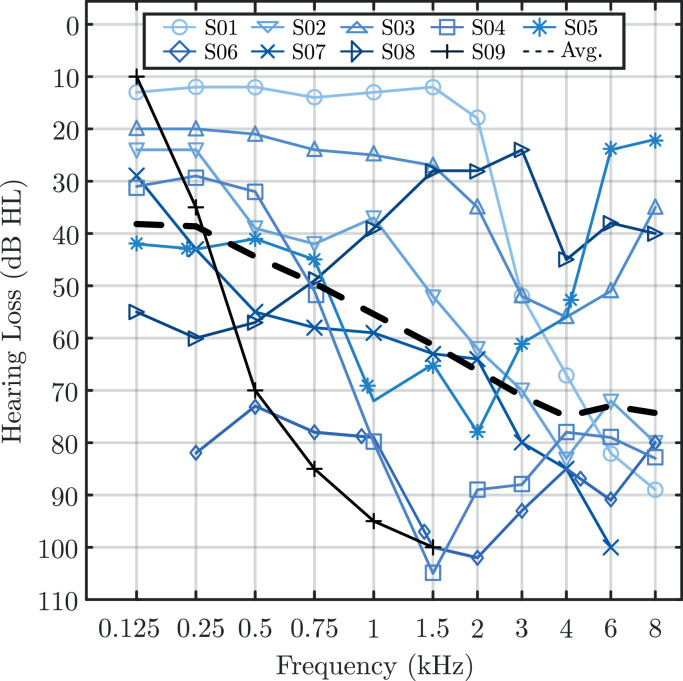
Air-conduction audiometric thresholds of the HA-side of the 10 study participants and average audiometric threshold across these participants (dashed line), which was used for the model. This figure is a reprint of Fig. 1 in Zedan et al. (2021) and is licenced under the Creative Commons licence CC BY 4.0.

Nine bimodal CI users, aged between 26 and 69 years old participated in the study of Zedan et al. (2021). All participants had their CI on the right side and the HA on the left side. Moreover, participants had a varying degree of hearing impairment on the contralateral side of the CI ranging from mild to severe hearing loss. Fig. 1 shows the audiograms of the participants. The average audiogram of the participants is shown using the black dashed line. This average audiogram will be used within the model to set the audiometric thresholds of the acoustic side in the hearing loss simulation.

**Fig. 2:**
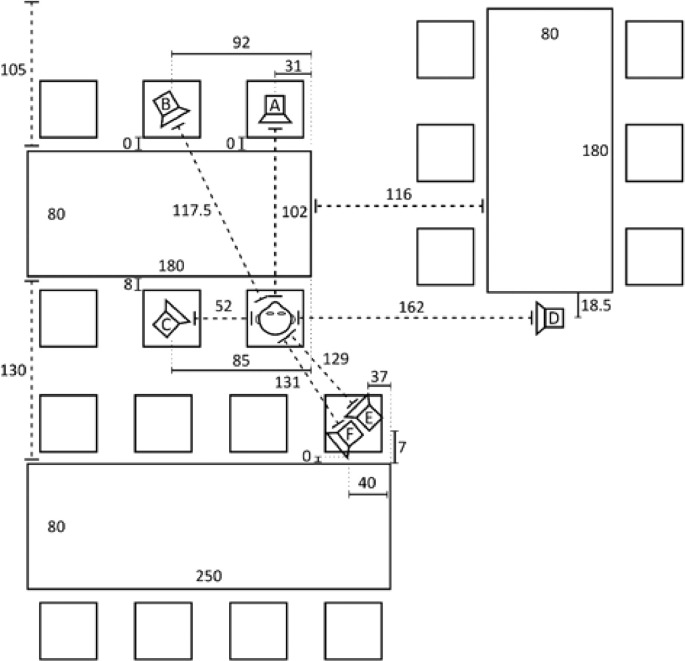
A diagram of the cafeteria scenario simulated using head-related impulse responses in both Zedan et al. (2021) and in this study. The listener is shown as a head symbol with the speech source situated in front of it from speaker A. Noise is omitted from positions A, through F depending on the noise scenario being simulated. This figure is a reprint of Figure 2 in Zedan et al. (2021) and is licensed under the Creative Commons licence CC BY 4.0 and is a reformatted reprint from Baumgärtel et al. (2015b).

*The Simulated Spatial Scenarios and Signals* The same simulated cafeteria scenario (see Fig. 2) used in Zedan et al. (2021) was used in the present study to produce the speech and noise material for the two models.

In this cafeteria scenario the listener is situated at a table surrounded by six sound sources. Four spatial scenarios, i.e., three single-source noise scenarios and one diffuse multi-talker babble noise scenario were reproduced. The three single-source noise scenarios are indicated by ${\text{S}}_{0}{\text{N}}_{-90}$, ${\text{S}}_{0}{\text{N}}_{0}$, and ${\text{S}}_{0}{\text{N}}_{+90}$, where the noise source was located to the left, front and right side of the listener from positions C, A and D, respectively. In those three scenarios, a single-source, stationary noise was used with the same long-term spectrum as the speech (’Olnoise’, Wagener et al. 1999). The fourth noise scenario was a 20-talker babble noise scenario (${\text{S}}_{0}{\text{N}}_{\text{20TB}}$), in which four intelligible, but uncorrelated speech signals were overlayed to one babble signal and five of these signals were each presented simultaneously on speakers (B, C, D, E, and F), representing a more complex, diffuse listening scenario. In all the simulated scenarios, the target speech was presented frontally from speaker A. The spatial scenarios ${\text{S}}_{0}{\text{N}}_{+90}$ and ${\text{S}}_{0}{\text{N}}_{\text{20TB}}$ are a subset of the spatial scenarios investigated also in Baumgärtel et al. (2015a). *Beamformer and HA Realization* The Master Hearing Aid (MHA, Grimm et al. 2006) was used to implement the ADM and MVDR, as was used in Baumgärtel et al. (2015b) and Zedan et al. (2021). Both the ADM and MVDR are spatial noise reduction algorithms that utilize the spatial separation between noise and speech to reduce the noise level.

The ADM is an adaptive beamformer (Elko and Pong, 1995) which is implemented monaurally on both sides of the listener. The denoised output of the ADM is written in equation (13) of Elko and Pong (1995):

(1) $$y\left(t\right)=c_{F}\left(t\right)-\beta c_{B}\left(t\right)$$

where β is a scalar that controls the blocking beam of the ADM. Moreover, $c_{F}\left(t\right)$ and $c_{B}\left(t\right)$ represent fixed front and back pointing beams that are generated from the microphones on the emulated device. In order to steer the blocking beam towards the loudest background noise, Elko and Pong (1995) adapted the scalar β to minimize the energy of the output using a gradient decent algorithm.

The MVDR is a binaural spatial noise reduction algorithm that uses the four input signals from the CI and HA microphones of the listener to produce a binaurally-denoised speech signal (Doclo et al., 2015). The MVDR implemented in this paper is a fixed beamformer and its output can accordingly be expressed as in equation (4) in Doclo et al. (2015):

(2) $$y=w^{H}x$$

where $w^{H}$ is the Hermitian of the MVDR filter impulse response and x is the input signal vector. The filter coefficients are calculated based on the head related transfer function (HRTF) of the listener in a given spatial scenario and the spatial coherence of the noise (Baumgärtel, Krawczyk-Becker, Marquardt, Völker, Hu, Herzke, Coleman, Adiloglu, Ernst, Gerkmann, Doclo, Kollmeier, Hohmann, Dietz, 2015, Doclo, Kellermann, Makino, Nordholm, 2015). Moreover, the MVDR aims at preserving the binaural quality of the target signal (i.e. speech) coming from the front. In this paper, as well as the work it is based on Baumgärtel et al. (2015b); Völker et al. (2015); Zedan et al. (2021), the HRTF is assume to be that of an anechoic room with the speaker to the front of the listener, and a diffuse noise field. Those assumptions facilitate the precalculation of filter coefficients, which results in a very simple and efficient (in terms of computational power) filter.

Both beamformers were compared to a no beamformer (NoBF) control condition. In line with how the patients were selected in Zedan et al. (2021), acoustic hearing with HA processing was always simulated on the left side and CI processing always on the right side. Moreover, the MHA was also used to simulate hearing aid processing on the left side with a multichannel dynamic range compressor (Herzke and Hohmann, 2005) using a modified CAMFIT (Moore et al., 1999) fitting formula, as described in Williges et al. (2019). CAMFIT was modified to focus on amplifying the residual hearing in CI users which is usually limited to low frequencies with an upper frequency limit of 5 kHz, and limiting the amount of maximum amplification to hearing losses below 90 dB HL.

### 2.2 FADE model

#### 2.2.1 Model overview

**Fig. 3:**
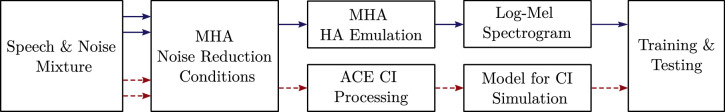
Block diagram of the model. All steps were performed within the framework of FADE, except for the steps labelled with Master Hearing Aid (Grimm et al. 2006, MHA). The signal at the CI and HA side are indicated by the red dashed line and blue solid line, respectively. Adapted and modified from Zamaninezhad et al. (2017). (For interpretation of the references to colour in this figure legend, the reader is referred to the web version of this article.)

The framework for auditory discrimination experiments (FADE) was used with modifications of Schädler et al. (2018), who evaluated different spatial noise reduction algorithms with HA users, and modifications of Zamaninezhad et al. (2017), who simulated SRTs of monaural combinations of acoustic and electric listening as present in CI users with remaining low-frequency acoustic hearing ipsilaterally. Both studies used simple feature concatenation and the same ASR backend. Fig. 3 shows a block diagram of the steps performed within FADE.

The first step is to generate a speech and noise mixture for the model. In this step, the head related impulse responses (HRIRs) used to create the virtual acoustics for the measurements in Zedan et al. (2021) were used to replicate the same acoustic scene and spatial scenarios (see Kayser et al. 2009. This generated 4-channel (with a rear and frontal channels on each side of the listener) speech and noise signals which were mixed at different SNRs with the desired number of test and training samples. Afterwards, the signals were processed using the Master Hearing Aid (MHA, Grimm et al. 2006) with the desired beamforming algorithms resulting in 2-channel speech and noise signals. Moreover, the MHA simulated multichannel dynamic range compression on the left side. As all subjects in Zedan et al. (2021) had their CI on the right and their HA on the left, this was replicated in the model. Furthermore, the same CI processing as done in Zamaninezhad et al. (2017) was replicated using GNU/Octave within FADE. Afterwards, features on the electric side were extracted as was done in Zamaninezhad et al. (2017), using a physiologically inspired auditory nerve spiking pattern model (Fredelake and Hohmann, 2012). The features on the acoustic side were extracted using a Log-Mel spectrogram, as done in Schädler et al. (2016b) and Hülsmeier et al. (2020) and concatenated to the electric features. Finally, FADE used the Hidden Markov Model Toolkit (HTK, Yoon et al. 2015) to train a Hidden Markov Model (HMM)-based speech recognizer. The trained speech recognizer was then used to replace the CI users, performing the speech test and predicting their speech recognition ability by estimating the SRTs, i.e. the lowest test SNR corresponding to a 50% correct word recognition rate.

Since the model was introduced extensively in other studies, descriptions will be kept compact in the following, however, highlighting the important steps of the model. There are five main steps in obtaining SRT predictions using FADE, corpus generation, sound processing, feature extraction, training, and recognition.

#### 2.2.2 Corpus generation

The first step in a FADE simulation is to generate training and testing files over a range of SNRs. This is necessary because training and testing needs to be performed over a range of SNRs to predict the SRT at which the hypothetical listener achieves 50% speech recognition. The SNR ranges can be pre-set in advance based on actual subject data or using trial and error. In this work, the ranges were chosen to cover the range where SRTs would be expected based on the clinical data provided by Zedan et al. (2021) and were then modified when the SRT was not in the specified range. Each simulation was performed across an SNR-range with 3 dB steps depending on the spatial scene and pre-processing condition used. Table 1 shows the simulated SNR-ranges used in training and testing.

**Table 1:** SNR-range of performed simulations in dB. Rows represent the spatial scene while the rows indicate the beamformer algorithm. NoBF: No beamforming; ADM: Adaptive differential microphone; MVDR: Minimum variance distortionless response.

Beamformer	${\text{S}}_{0}{\text{N}}_{-90}$	${\text{S}}_{0}{\text{N}}_{0}$	${\text{S}}_{0}{\text{N}}_{+90}$	${\text{S}}_{0}{\text{N}}_{\text{20TB}}$
NoBF	–13 to 5 dB	–9 to 6 dB	–13 to 5 dB	–10 to 8 dB
ADM	–18 to 0 dB	–9 to 6 dB	–18 to 0 dB	–16 to 2 dB
MVDR	–24 to -6 dB	–9 to 6 dB	–21 to -3 dB	–18 to 0 dB

After defining the ranges at which the simulations needed to be performed, the desired speech and noise signals were mixed according to the specified SNR range for each condition and were processed using MHA as will be discussed below.

As in Zedan et al. (2021), the German matrix sentence test (OLSA, Wagener et al. 1999) was used as target speech. 120 sentences from the OLSA were mixed with noise and used for each SNR point. Each of the 120 sentences is composed of 5 words: a name, a verb, a number, an adjective, and an object, each with ten possible words. For example: “Peter bekommt vier grne Autos” which translates into: “Peter receives four green cars”. Each possible word within the 120 sentences was uttered 12 different times, giving the speech some intrinsic variability. The 120 sentences were mixed with different instances of the desired noise for a total of nine times for each spatial scenario, each beamformer, and SNR. Eight of the mixtures (i.e. 960 sentences) were used to train the model for one SNR condition, and one mixture (i.e. 120 sentences) was used in testing for another SNR.

#### 2.2.3 Sound processing and feature extraction

This section discusses the reason behind the choice of the features. It also summarizes the processing steps from noisy speech material that was processed using the room simulation, beamformer (and hearing aid simulation on the left side) into features used as input in the ASR system for both HA and CI side. *The Choice of Features* As the goal of this study is to model the SRTs of the measured bimodal CI users using a functional model, a proven and functional approach was followed to achieve that goal using the FADE model. FADE was used mostly with phenomenological feature extraction models (Hülsmeier, Warzybok, Kollmeier, Schädler, 2020, Kollmeier, Schädler, Warzybok, Meyer, Brand, 2016, Schädler, 2015, Schädler, Hülsmeier, Warzybok, Kollmeier, 2020, Schädler, T, Kollmeier, 2018, Schädler, Warzybok, Ewert, Kollmeier, 2016). In the case of modelling CI users speech-in-noise performance, only biophysical models were used (Jürgens, Hohmann, Bchner, Nogueira, 2018, Zamaninezhad, Hohmann, Bchner, Schädler, Jürgens, 2017). Zamaninezhad et al. (2017) used a particular combination of biophysical models (Fredelake et al., 2012 for the electric stimulation and Meddis, 2006 for the acoustic stimulation), because their speech intelligibility prediction was modelled in the same ear, which means that both electric and acoustic features must be present in the same auditory nerve. It is notable, that their modelled acoustic-only SRTs were relatively poor, i.e., never better (lower) than +7 dB SNR. Preliminary model attempts with the Meddis model (Meddis, 2006) showed that much better SRTs were not being achieved even in NH mode. That is a fact that was also shown in Clark et al. (2012), whose best SRTs were just above 0 dB SNR. In the present study with bimodal CI users, the contralateral acoustic hearing is relatively well preserved, much better than the ipsilateral acoustic hearing in the patients used to model in Zamaninezhad et al. (2017). Therefore, this paper uses one of the simplest, but successful known features for the FADE model for the present study, which are log-Mel spectrogram features on the acoustic side and the temporally and spectrally integrated neuronal activity on the electric side. Since both features (electric and acoustic) are representations of log-spaced frequency channels as a function of time, they are highly comparable even though they originate in quite different model approaches (biophysical and phenomenological).

*Hearing Aid Side (left)* On the HA side, the input signal was first convolved with the impulse response of the ER-4 in-ear headphones to eardrum provided by Denk and Kollmeier (2021). This was done because the ER-4s were used to substitute the HA and deliver the MHA processed acoustic signal to the bimodal CI user in Zedan et al. (2021). Afterwards, the feature extraction was performed as described in Schädler et al. (2016b) which is summarized below:

The Log-Mel spectrogram of the input audio was calculated. This starts by downsampling the audio signal to a sampling frequency of 16 kHz and then dividing it into frames with a frame length of 25 ms with 10 ms frame shifts. The spectrum was then calculated using a fast Fourier transform (FFT) after applying a normalized Hamming window to each frame. Afterwards, a 31-channel triangular Mel filter-bank was used to weigh and sum the FFT output to obtain the Mel-frequency bin values. Finally, the base-10-logarithm of the Mel-frequency bins was used as features. This resulted in 31 log-Mel coefficients for each frame of the input acoustic signal (Schädler et al., 2016b).

In order to simulate the hearing loss, the hearing threshold corresponding to the hearing loss was determined by adding the hearing loss level in dB HL to the hearing loss threshold defined in the ISO 226 (2003) standard loudness curves in dB SPL. The hearing loss values were calibrated to ear drum values as done in Hülsmeier et al. (2021). Finally, the hearing threshold in dB SPL was subtracted from the Log-Mel Spectrogram. Values below 0 dB SPL were replaced with random samples from a white Gaussian distribution with a mean of zero and standard deviation of one, simulating the loss of audibility. Thus, only values exceeding the individual hearing threshold could be used by FADE for the recognition process (Schädler et al., 2016b).

*Cochlear Implant Side (right)* Following the work done in Jürgens et al. (2018) and Zamaninezhad et al. (2017), feature extraction on the CI side was done using the model of Fredelake and Hohmann (2012). The number of auditory nerve cells used in this simulation was set to 2200 and the spatial spread constant was set to 1 mm (see below), otherwise, the same model parameters as in Zamaninezhad et al. (2017) were used. The following sections are based on Zamaninezhad et al. (2017) and Fredelake and Hohmann (2012), see those studies for more details. *CI Coding Strategy* Feature extraction on the CI side started by generating the electric stimulation pattern using the desired CI coding strategy. The ACE coding strategy (Nogueira et al., 2005) used in this work stimulates the electrodes corresponding to the N=8 channels with the largest amplitudes out of M = 22 channels within a time frame of 1.1ms. The channel-specific stimulation rate was 900 pulses per second (pps) which resulted in a total stimulation rate of 7200 pps (900 x 8). In each cycle, 128 audio samples were analysed by ACE resulting in analysis frames with a length of 8 ms (due to the input sampling frequency of 16kHz) with an overlap of 6.89 ms (8 ms - 1/900 pps) between frames, which is a direct result of the stimulation rate. The frames were Hann-windowed, and their spectrum was calculated using an FFT with a size equal to the Hann window size of 128 samples. The FFT spectrum was then used to create an FFT filter bank with a number of band-pass filters equal to the number of CI electrodes M by combining the FFT bins of the FFT output. The Hilbert transform was used to obtain the envelope of each channel. Then, the N channels that corresponded to the highest amplitudes generated the CI firing pattern at the electrode for each cycle. Finally, the same values used in Zamaninezhad et al. (2017) for threshold levels (THL) and most comfortable levels (MCL) were used to generate the current values. The eight electrodes selected for stimulation were stimulated in base-to-apex order, i.e., starting with the electrode that corresponds to the channel with highest center frequency down to the one with the lowest center frequency. More details regarding the FFT filter bank and ACE coding strategy used can be found in Nogueira et al. (2005). *The Auditory Model for Electrical Stimulation* The features on the CI side were generated using the Fredelake and Hohmann (2012) model which simulates electrically stimulated auditory nerve cells using an integrate-and-fire model. Hereby, the electrode array was simulated with 0.75 mm spacing between electrodes ranging from 8.125 mm to 23.875 mm from the apex inserted in a cochlea of 35 mm length. The cochlea was assumed to consist of 2200 nerve cells that were equidistantly distributed over this length. Spatial spread of the electric current inside the cochlea was simulated as a double-sided one-dimensional exponentially decreasing function. Multiplying the value of the spatial spread function with the current amplitude, gives the current value at the position of the simulated nerve cell. While Zamaninezhad et al. (2017) simulated a range of spatial spread constants, the present study uses a distance constant λ = 1 mm, indicating a 36.8% drop in the current amplitude 1 mm away from the electrode.

Each nerve cell was simulated as an integrate-and-fire neuron including refractory effects, membrane noise, latency and jitter effects. For details about modeling the nerve cells see Fredelake and Hohmann (2012). Those processes are repeated for each electric stimulus generated by the simulated CI and for all nerve cells in a recursive manner, resulting in a vector of the action potential event times and the cells responsible for those action potentials.

Afterwards, the model generated what is called an “internal representation” (IR) which represents the result of the central auditory processing and which was used as features for the automatic speech recognizer. This was done through both temporal and spatial integration of the action potentials. Spatial integration was done by summing the action potentials of cells surrounding the electrodes. In the model, the action potentials of the nerve cells closest to an electrode were summed, separated by the midpoint between the electrodes and resulting in 22 spatial groups in total. In other words, a group of neural cells was spread over a 0.75 mm length centred on each electrode. The temporal integration was done using two filters, a non-recursive Gaussian filter simulating temporal resolution reduction in CI users and a recursive integrator acting as a low-pass filter. The model was also able to simulate the central auditory system degradation effects by multiplying the generated IR with white Gaussian noise. As a multiplicative noise, the mean was set to 1, and in this work, the variance was set to 0.1. Finally, the mean and the variance of the features were normalized to 0 and 1, respectively.

*Feature Combination* To use the generated features to train and test the speech recognizer, they were simply concatenated. Therefore, the resulting features from the HA and CI sides, with respective dimensionality of 31 and 46, are combined, resulting in a feature dimension of 77. No other binaural processing was used to combine the data simulating binaural processing before the automatic speech recognizer stage.

#### 2.2.4 Training and recognition

After feature extraction and combination, training was done to estimate two important parameters of the model, the state transition probabilities of the Markov chain, and state observation distribution parameters. Each word was modelled using eight emitting states, while the silence, beginning and end of a model were represented using six, three and three emitting states, respectively. The start and stop models handled random features on the start and end of recordings and were right to left HMMs like the models representing words. A silence model was used to represent the silence before/after each sentence. As for observations, a GMM composed of a single component was used, resulting in a Gaussian distribution with a dimensionality matching that of the feature vectors’ dimensionality. Afterwards, each trained model was tested on the complete range of SNRs, giving a squared matrix of speech recognition results in percentage points with training and testing SNRs as the two dimensions. The results were then interpolated and the lowest testing SNR resulting in 50% correct word recognition was selected as the predicted SRT.

### 2.3 BSIM: Bimodal speech intelligibility model

The BSIM model (Beutelmann et al., 2010) was extended in Williges et al. (2015) and Zedan et al. (2018) to predict SRTs of vocoder experiments simulating bimodal CI users and is used in this study as short-term BSIM2010 version to predict the SRTs of the actual bimodal CI users in (Zedan et al., 2021). In agreement with Beutelmann et al. (2010) and Zedan et al. (2018), the speech matrix test material was replaced by Olnoise, as it has the exact same long-term spectrum as the speech and because BSIM as a macroscopic speech intelligibility model only regards the long-term spectrum. Moreover, the same spatial scene simulation using HRIRs, beamformer and HA signal processing steps using the MHA as performed in Zedan et al. (2021) and as in the FADE model above are done. However, BSIM requires separate speech and noise signals, to calculate SNRs which are the basis of the prediction. Since the beamformers used here are adaptive and would show different adaptation when only speech and only noise was presented to them and the subsequent HA processing on the left side is nonlinear, the disentangled speech and noise signals at the output of the MHA were obtained using the Hagerman-Olofsson method (Baumgärtel, Krawczyk-Becker, Marquardt, Völker, Hu, Herzke, Coleman, Adiloglu, Ernst, Gerkmann, Doclo, Kollmeier, Hohmann, Dietz, 2015, Hagerman, Olofsson, 2004). Using this method, the input speech and noise signals are mixed twice, once by adding them and once by subtracting the noise from the speech signal. Each mixture is processed with MHA using the same configuration used in the model simulations. At the output, adding the two mixtures or subtracting them from each other and dividing the resulting output by two gives the pure processed speech or noise signals, respectively, for each side.

Next, the noise and speech signals were processed with device-specific signal processing on the respective listener side. On the right side, the same CI processing steps for the ACE coding strategy (Nogueira et al., 2005) were used to process the input signals. This was followed by a spatial current spread simulation and a Gammatone filter bank which is used to auralize the signal (Jürgens et al., 2021). On the left side, hearing loss is simulated by reducing the audibility of the input signals based on the same average audiogram shown in Fig. 3.

In addition to the monaural signals on the left and right sides of the listener, BSIM produces a binaurally processed signal using an equalization cancellation (EC) step (Durlach, 1963) which enables it to predict the binaural hearing benefit seen in NH listeners (Beutelmann and Brand, 2006). Out of these three signals and for each frequency band of the model separately, the best SNR is used for the prediction of the SRT using the speech intelligibility index (SII, ANSI 1997) algorithm. As acoustic (HA side) and electric (CI side) modes of hearing are combined and scenarios may result in one side (e.g., the acoustic side) showing better SNRs at low frequencies, but the other side (the electric) better SNRs at high frequencies, a function combining these SNRs was used (”electro-acoustic weighting function”), the same as used in Zedan et al. (2018) to get the average SNR across frequency bands. Once the SNR was obtained, an iterative process was used to find the SRT. In this process the input SNR is iteratively changed by adding or subtracting a broadband gain value to/from the speech spectrum until the SII output matches a given reference SII value. Two different SII values are preset for the CI and acoustic sides (Williges et al., 2015). As was done in Zedan et al. (2018), the SII reference value (SII = 0.26) for the acoustic side is set to correspond to the SRT a NH subject listening to one ear only (i.e. monaurally) in a ${\text{S}}_{0}{\text{N}}_{0}$ spatial scenario. Similarly, the CI SII value (SII = 0.42) is calibrated to match the average SRT of the CI users of Zedan et al. (2021) ${\text{S}}_{0}{\text{N}}_{0}$ spatial scenario listening with the CI only. In other words, the model was set match to empirical reference data.

### 2.4 Model evaluation and comparison

Table 2 shows a comparison between the BSIM and FADE models.

**Table 2:** Summary of comparing BSIM and FADE. BSIM: Binaural speech intelligibility model (Beutelmann, Brand, Kollmeier, 2010, Zedan, Williges, Jürgens, 2018); FADE: Framework for Auditory Discrimination Evaluation (Schädler et al., 2016b).

Model	BSIM	FADE
Back-end	Speech intelligibility index	Automatic speech recognizer
Advantages	Computationally efficient, can use binaural processing	Speech-noise-mixtures as input, suitable for non-linear and adaptive processing
Disadvantages	Needs isolated speech and noise signals, needs empirical reference data	Computationally complex, requires a-priori knowledge about sentence structure.

Both BSIM and FADE are effective models, however, FADE is a more comprehensive framework that offers more flexibility and does not require empiricial reference data for its prediction.

#### 2.4.1 Calculating instrumental SNR beamformer benefit

As a comparison to the beamformer algorithm benefit seen in the measured and modelled results, the instrumental SNR improvement was calculated by subtracting the input SNR from the output SNR (Greenberg et al., 1993). For the input signal, the frontal microphone signal from each side was used to calculate the input signal SNR. For the output signal, the broadband SNR improvement from using the beamformers was measured using the Hagerman-Olofsson method (Baumgärtel, Krawczyk-Becker, Marquardt, Völker, Hu, Herzke, Coleman, Adiloglu, Ernst, Gerkmann, Doclo, Kollmeier, Hohmann, Dietz, 2015, Hagerman, Olofsson, 2004). The resulting speech and noise signals can then be used to calculate the output SNR. For each spatial scenario and each beamformer condition, two instrumental SNRs were calculated, one corresponding to each side of the listener.

#### 2.4.2 Statistical analysis

Pearson’s correlation coefficient and root-mean-square (RMS) errors were used to evaluate the accuracy of the FADE and BSIM SRT predictions against the medians of measured SRTs of bimodal CI users in Zedan et al. (2021). Moreover, these measures were used to compare the predicted SRT-benefit in FADE and BSIM as well as the instrumental SNR improvements against the measured SRT benefit achieved by the CI subjects. The data points used in the correlation were the SRT benefit in each spatial scenario and each beamformer condition.

## 3 Results

**Fig. 4:**
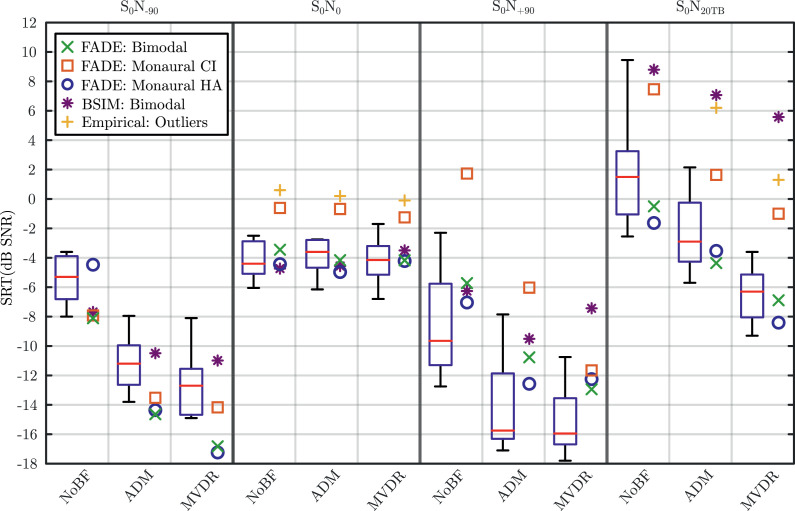
Whisker-boxplots of the measured SRTs (Zedan et al., 2021) and the FADE model predictions of bimodal CI, monaural CI, and monaural HA hearing shown as green “x” markers, red squares and blue circles, respectively. Moreover, BSIM predictions are shown in purple “*” markers. The four panels of the figure separated by dashed lines show each of the spatial scenarios (${\text{S}}_{0}{\text{N}}_{-90}$, ${\text{S}}_{0}{\text{N}}_{0}$, and ${\text{S}}_{0}{\text{N}}_{+90}$, and ${\text{S}}_{0}{\text{N}}_{\text{20TB}}$). Each panel show the whisker-boxplots of measured data and model predictions for each of the beamformer conditions (NoBF, ADM, and MVDR). (For interpretation of the references to colour in this figure legend, the reader is referred to the web version of this article.)

Fig. 4 shows the FADE and BSIM SRT predictions (different marker symbols) alongside the measured data reported in Zedan et al. (2021) shown as box-and-whisker plots. The figure displays four panels, one for each of the spatial scenarios. Each panel displays measured and modelled SRTs obtained through the three beamformer conditions as labelled at the bottom of the figure.

In general, the SRTs predicted by the FADE and BSIM simulations (green ’x’ and purple ’*’ markers, respectively) follow the speech recognition patterns of the subjects’ empirical measurements. Both FADE’s and BSIM’s predictions of the medians of measured SRTs in the co-located speech and noise scenario (${\text{S}}_{0}{\text{N}}_{0}$) are fairly accurate. However, FADE’s predictions were lower than the measured SRTs (better) in ${\text{S}}_{0}{\text{N}}_{-90}$, which overestimated the speech recognition ability of bimodal CI users while underestimating it in the ${\text{S}}_{0}{\text{N}}_{+90}$ scenario. In the ${\text{S}}_{0}{\text{N}}_{\text{20TB}}$, FADE predictions were only slightly lower than measured SRTs.

On the other hand, BSIM predictions tended to predict higher SRTs (worse), underestimating the speech recognition ability of measured bimodal CI users. The largest differences were seen in the ${\text{S}}_{0}{\text{N}}_{\text{20TB}}$ scenario where BSIM predictions were considerably higher than the measured SRTs.

To study the way FADE combines information from the CI and HA sides, FADE was also used to predict the SRT values of the different spatial scenarios and beamformer algorithms for monaural CI and monaural HA conditions, even though the study of Zedan et al. (2021) did not include these monaural conditions. In a clinical setting this would correspond to switching off either the CI or the HA in the spatial scenario. The model predictions for monaural CI and HA hearing are also shown in Fig. 4 as orange squares and blue circles, respectively. The SRT-predictions for the monaural CI simulations were consistently higher (poorer) than those of the monaural HA condition except for the ${\text{S}}_{0}{\text{N}}_{-90}$ scenario with NoBF, where the noise is being presented from the HA side. This difference vanished when beamformers were used. Moreover, FADE predictions in the bimodal condition (green crosses) were close to the better performing side. The largest differences between the bimodal condition and best performing side were observed in cases where the difference between the monaural CI and monaural HA simulation predictions were highest. For example, this was mostly pronounced in ${\text{S}}_{0}{\text{N}}_{+90}$ and in ${\text{S}}_{0}{\text{N}}_{\text{20TB}}$.

**Fig. 5:**
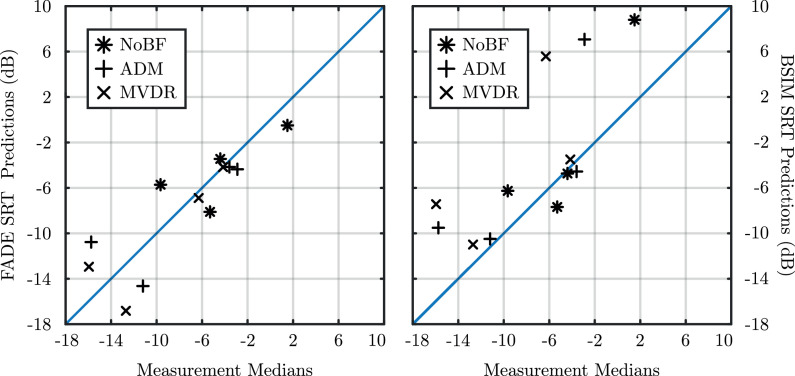
FADE (left plot) and BSIM (right plot) SRT predictions versus medians of measured data. The straight line indicates perfect a correlation coefficient of 1.00. The NoBF, ADM, and MVDR conditions are indicated by the signs *, +, and x, respectively.

To further assess the accuracy of the models, scatter plots between the medians of the measured SRTs and models’ predictions for the bimodal condition are shown in Fig. 5. Points laying on the blue diagonal line would mean that there was a perfect prediction, while star, plus and cross signs represent NoBF, ADM and MVDR predictions vs measurement medians, respectively.

Overall, a high Pearson’s correlation coefficient between the medians of measured SRTs and the FADE’s predictions (left panel) of r = 0.85 (p<0.001) and an RMSE of 2.8 dB was observed. The predictions were less correlated for lower (more negative) SRTs which correspond to the ${\text{S}}_{0}{\text{N}}_{-90}$ and ${\text{S}}_{0}{\text{N}}_{+90}$ scenarios. On the other hand, BSIM (right panel) had a lower overall correlation coefficient of 0.73 (p<0.01) and a higher RMSE of 6.0 dB. These considerable differences can be largely attributed to the large bias in the ${\text{S}}_{0}{\text{N}}_{\text{20TB}}$ condition. Therefore, Table 3 shows a breakdown of the correlation and RMSE differences between FADE and BSIM for each of the spatial scene scenarios.

**Table 3:** Pearson’s correlation coefficients for FADE and BSIM. The overall correlation was calculated over all spatial scenarios and beamformer conditions while spatial scene specific correlations were calculated over beamformer algorithms. ${}^{*}$, ${}^{**}$ and ${}^{***}$ indicate a statistically significant correlation with a p-value of <0.05, <0.01 and <0.001, respectively, between the measured SRT improvements and the compared algorithms.

Model	Correlation coefficient					RMSE (dB)				
	${\text{S}}_{0}{\text{N}}_{-90}$	${\text{S}}_{0}{\text{N}}_{0}$	${\text{S}}_{0}{\text{N}}_{+90}$	${\text{S}}_{0}{\text{N}}_{\text{20TB}}$	Overall	${\text{S}}_{0}{\text{N}}_{-90}$	${\text{S}}_{0}{\text{N}}_{0}$	${\text{S}}_{0}{\text{N}}_{+90}$	${\text{S}}_{0}{\text{N}}_{\text{20TB}}$	Overall
FADE	$1.00^{*}$	−0.73	0.96	$1.00^{*}$	$0.85^{***}$	3.5	0.6	4.1	1.5	2.8
BSIM	$1.00^{*}$	−0.08	0.76	$1.00^{*}$	$0.73^{**}$	1.7	0.7	6.4	9.9	6.0

Both models show no significant correlation in the ${\text{S}}_{0}{\text{N}}_{0}$ and ${\text{S}}_{0}{\text{N}}_{+90}$ conditions. As expected, a large portion of the large RMSE difference between FADE and BSIM can be explained by the highly elevated predictions by BSIM in the ${\text{S}}_{0}{\text{N}}_{\text{20TB}}$ scenario as is reflected in the correlation coefficient and RMSE values. Nonetheless, BSIM had a lower RMSE than FADE in the ${\text{S}}_{0}{\text{N}}_{-90}$ spatial scene.

### 3.1 Algorithm benefit

**Fig. 6:**
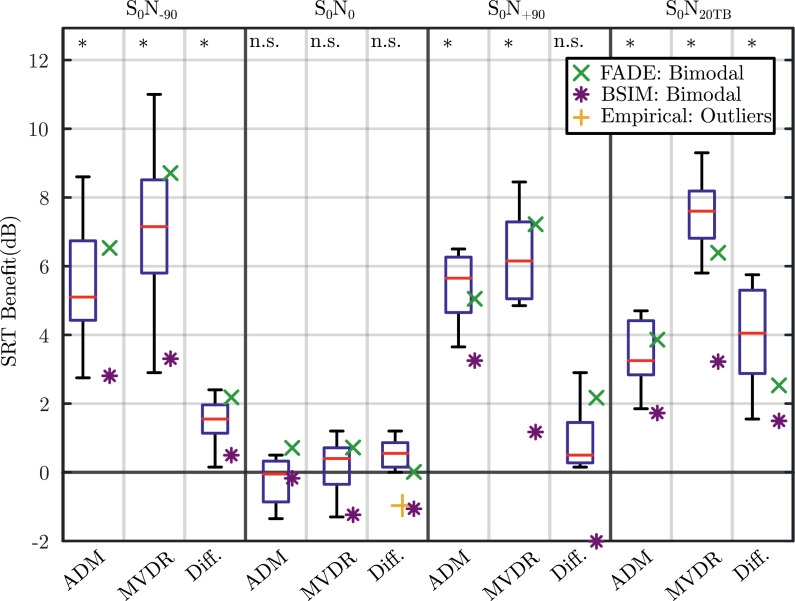
Whisker-boxplots of the measured SRT benefit from using beamformers (Zedan et al., 2021). FADE and BSIM predictions are shown as green “x”, and purple “*” markers, respectively. The four panels of the figure separated by dark lines show each of the spatial scenarios (${\text{S}}_{0}{\text{N}}_{-90}$, ${\text{S}}_{0}{\text{N}}_{0}$, and ${\text{S}}_{0}{\text{N}}_{+90}$, and ${\text{S}}_{0}{\text{N}}_{\text{20TB}}$). Each panel shows the SRT benefit using the ADM and MVDR over NoBF, and the difference in advantage of using the MVDR over the ADM (indicated by ADM, MVDR, and Diff., respectively). ${}^{*}$ indicates a statistical significance with a p-value of <0.001 between the compared beamformer conditions. (For interpretation of the references to colour in this figure legend, the reader is referred to the web version of this article.)

Fig. 6 shows a comparison between algorithm benefit measured in the empirical study of Zedan et al. (2021) and algorithm benefit predicted by the models.

The benefit of using the beamformers (i.e. the difference between the NoBF and either the ADM or the MVDR within the same spatial scenario) was very well predicted by FADE. The predicted benefit of using the beamformers was larger in the ${\text{S}}_{0}{\text{N}}_{-90}$ scene compared to ${\text{S}}_{0}{\text{N}}_{+90}$, as also observed in measured SRTs. Moreover, the MVDR was predicted to perform consistently better than the ADM in all conditions where noise was not co-located with speech. FADE was also able to predict the much larger difference in benefit in ${\text{S}}_{0}{\text{N}}_{\text{20TB}}$ compared to ${\text{S}}_{0}{\text{N}}_{-90}$ and ${\text{S}}_{0}{\text{N}}_{+90}$. On the other hand, BSIM consistently underestimated the benefit using the beamformers. In some cases, BSIM predicted the MVDR to perform as well as the ADM, or even worse, as seen in ${\text{S}}_{0}{\text{N}}_{+90}$.

Finally, the broadband SNR benefit due to beamformer algorithms is shown in Table. 4.

**Table 4:** Instrumental SNR benefit calculated by taking the difference between input and output broadband SNR. In the MVDR vs. ADM case, the difference was taken between MVDR and ADM algorithm improvement.

Beamformer	Left (HA) side				Right (CI) side			
	$S_{0}N_{-90}$	$S_{0}N_{0}$	$S_{0}N_{+90}$	$S_{0}N_{20\text{TB}}$	$S_{0}N_{-90}$	$S_{0}N_{0}$	$S_{0}N_{+90}$	$S_{0}N_{20\text{TB}}$
ADM vs. NoBF	4.2	0.0	4.6	2.2	5.2	0.0	4.8	5.0
MVDR vs. NoBF	11.3	–0.1	4.8	6.3	5.6	0.0	6.9	7.6
MVDR vs. ADM	7.0	0.0	0.1	4.1	0.4	0.0	2.1	2.6

The SNR improvement is shown for each side, separately. Note that the algorithm benefit varies in large amounts between the left and right sides for the same spatial situation and same beamformer. As can be see, there is an absolute difference of 2,8 dB and 5,7 dB between the left and right sides in the $S_{0}N_{20\text{TB}}$ and $S_{0}N_{-90}$ spatial scenarios, respectively.

Table 5 shows the Pearson’s correlation coefficients between the measured algorithm benefit and different model predictions in the ADM and MVDR beamformer conditions. The instrumental SNR improvements were calculated by averaging the right and left side SNR improvements shown in Table 4.

**Table 5:** Pearson’s correlation coefficient between the measured SRT benefit and FADE, BSIM and instrumental SNR improvements (iSNR) for the ADM and MVDR. ${}^{*}$ and ${}^{**}$ indicate a statistically significant correlation with a p-value of <0.01 and <0.001, respectively, between the measured SRT improvements and the compared algorithms.

Model	iSNR	FADE	BSIM
r	$0.97^{**}$	$0.96^{**}$	$0.91^{*}$
RMSE	0.7	1.0	2.9

As shown in the table, FADE has a higher SRT-benefit correlation coefficient than BSIM and is comparable to that of the instrumental SNR improvements. Moreover, RMSE values for FADE were lower than those of the BSIM and were very close to those of the instrumental SNR measurements.

## 4 Discussion

The present study compared the ability of a machine learning-based model (FADE) and the speech-intelligibility-index based model (BSIM) to predict the SRTs of bimodal CI users reported in Zedan et al. (2021) and the benefit they experience from using beamformers. FADE is a speech intelligibility prediction framework (Schädler et al., 2016b) based on an ASR backend and can predict the SRTs in various noise types independent of any empirical reference data. FADE was extended in this study based on the electroacoustic CI model reported in Zamaninezhad et al. (2017) using simple feature concatenation to combine electric and acoustic internal representations. BSIM was introduced in Beutelmann et al. (2010), which was extended for predicting vocoder experimental data simulating electroacoustic CI users in Williges et al. (2015) and simulating bimodal users in Zedan et al. (2018). The exact same three spatial scenarios with a single stationary noise interferer and a diffused 20-talker babble noise scenario containing more fluctuations, and the same beamformer and hearing aid processing were used in the models, as in the experimental study (Zedan et al., 2021).

### 4.1 FADE predictions

FADE was able to perform considerably well in predicting the absolute SRTs in all the spatial scenarios and beamformer conditions (r = 0.85, p<0.001, RMSE = 2.8 dB). This result joins other studies showing that FADE is particularly well suited for speech intelligibility predictions with NH (Schädler et al., 2015), hearing-impaired (Kollmeier et al., 2016), CI users (Jürgens et al., 2018), ipsilateral electro-acoustic CI users (Zamaninezhad et al., 2017), and hearing-aid users (Schädler et al., 2020), and outperforms other models, such as the speech intelligibility index (Schädler et al., 2016a). Given the relatively complex nonlinear HA and CI processing resulting in restrictions and enhancements of speech reflected in the features, FADE learns to optimally assign these features to word and phoneme classes, however, regarding their speech-intrinsic variability (Schädler et al., 2015). This machine-learning approach outputs then ”optimal” speech intelligibility, given the restrictions in the features. Using concatenation, FADE seems to concentrate on the most salient speech features across electric and acoustic feature compartments and seems to largely ignore spectro-temporal patterns that do not help in discriminating speech tokens.

A comparison of the FADE-predicted SRTs obtained for bimodal CI and HA usage to the predicted SRTs with monaural CI and monaural HA usage (see Fig. 4, squares and circles) reveals that bimodal SRTs (crosses) were never significantly better (i.e., lower) than the best monaural SRT, which indicates the absence of combined benefit (Jürgens et al., 2021) in the model. Differences between the best monaural and bimodal SRTs were less than 2 dB, indicating better-ear-listening (Williges et al., 2019), which can most likely be attributed to the poor processing ability of especially interaural time differences (Angermeier, Hemmert, Zirn, 2021, Francart, McDermott, 2013, Laback, Egger, Majdak, 2015, Zirn, Angermeier, Arndt, Aschendorff, Wesarg, 2019) in individuals using a CI on at least one side. Lack of combined benefit in the model may be interpreted as that the most salient speech features used by the model for speech recognition seem to be limited to either exclusively the electric or the acoustic feature-part, but do not seem to combine across the concatenated features.

Furthermore, the monaural HA side was consistently the better performing side except for the $S_{0}N_{-90}$ scenario with no beamformer. This can be explained by the fact that the simulated subject group in Zedan et al. (2021) included subjects with a large variation in hearing loss on the HA side which resulted in only a moderate hearing loss audiogram as an average. When beamforming is used, the better performance seen in the monaural CI condition in $S_{0}N_{-90}$ diminishes. This may be caused by asymmetrical algorithm benefit in this scenario, specifically, a higher iSNR improvement (11.3 dB) on the HA side compared to the CI side (5.6 dB) when using the MVDR.

The prediction of algorithm benefit by FADE was also highly accurate (r = 0.96, p<0.001, RMSE = 1.0 dB), and SRT-improvements due to using the ADM and MVDR were on scale with the experimental data (Zedan et al., 2021) and with instrumental SNR-improvements averaged across ears (r = 0.97, p<0.001, RMSE = 0.7 dB). That was despite the already mentioned smaller differences between measured and predicted absolute SRTs. As in the experimtal data, FADE predicted higher SRT-improvements when using the binaurally implemented MVDR compared to the monaurally implemented ADM in the $S_{0}N_{20\text{TB}}$ scenario. The benefit of using the MVDR over the ADM can be attributed to the fact that the ADM was designed for single noise source scenarios and the MVDR was designed for diffused noise scenarios (Baumgärtel et al., 2015b). Moreover, as discussed in Baumgärtel et al. (2015a), the higher benefit from using the MVDR compared to the ADM seen in bilateral CI users, but not bilateral HA users or normal hearing subjects (Völker et al., 2015), can again be related to the lack of binaural processing in CI users. Since they are missing internal binaural processing, external binaural processing using MVDR provides higher benefit. The ability to replicate such results in terms of both, absolute SRTs and SRT-benefits is an important aspect of the model that may be critical for analysing for analysing and developing new spatial noise reduction algorithms for bimodal CI users. When it comes to pure SRT-benefit predictions, however, the iSNR-improvement averaged across ears seems to be a much simpler model to use than FADE.

In this work, FADE simply concatenated the features from the CI and HA sides without introducing any binaural processing on the fine time structure, which is supposed to take place in the NH binaural system (Beutelmann and Brand, 2006). If bimodal CI users had some ability of fine-structure binaural processing, this may have explained the prediction offsets by FADE in the $S_{0}N_{+90}$ and $S_{0}N_{-90}$ scenarios. Evidence for that is predominantly found in carefully controlled laboratory measurements (Francart et al., 2008). However, studies employing less controlled commercial speech processors, couldn’t replicate these benefits for speech processing (Balkenhol, Wallhusser-Franke, Rotter, Servais, 2020, Dieudonn, Francart, 2020, Williges, Wesarg, Jung, Geven, Radeloff, Jürgens, 2019). Introducing and validating a binaural processing strategy in a normal-hearing setting for FADE may offer an insight into possible missing binaural processing in bimodal CI users. Moreover, Zamaninezhad et al. (2017) showed that they were able to individualize their model and predict the SRTs of individual subjects by varying the model parameters. They varied the number of simulated nerve cells, spatial spread constant, multiplicative internal noise variance and upper bounds for residual acoustic frequency which is specific to electroacoustic CI subjects. The FADE model presented in this paper can also be used to predict individual bimodal CI subjects by varying the audiogram used in simulating the hearing loss and the fitting of the hearing aid formula to the desired individual.

### 4.2 BSIM predictions

While BSIM was able to perform well in predicting the absolute SRTs of bimodal CI users in three spatial scenarios with a single source stationary noise source, it struggled in the 20-talker babble noise scenario, and in predicting the benefit due to using the beamformer algorithms. BSIM was particularly designed for modeling binaural speech processing in NH listeners ”effectively” simulated by the EC-stage in the model that uses the fine temporal structure of right and left signals to improve the SNR. Zedan et al. (2018) used BSIM similarly to here, in order to predict SRTs collected with vocoder experiments simulating single-sided deaf CI users and bimodal CI users. They found out that, although the EC-stage was available to the model, the vocoder on one side erased the ability of using the fine temporal structure to perform the EC-algorithm, and consequently, the model output was relying exclusively on the best SNR from either the right or left signal, but never from the binaurally processed signal. This lack of binaural processing benefit was also observed in BSIM of the present study, despite the availability of its binaural processing stage, in the form of the EC algorithm (Durlach, 1963).

While BSIM had higher RMSE values and correlated less with the measured data, it is notable that a large part of the difference in the BSIM values can be explained by the substantially higher SRT predictions of the BSIM model in the $S_{0}N_{20\text{TB}}$ scenario (RMSE = 9.9 dB). A possible explanation might be that the 20-talker babble noise used in this scenario was more fluctuating than the stationary Olnoise used in the other three scenarios (Beutelmann et al., 2010). Although the short-term version of BSIM was used, possible “listening-in-the-dips” effects might not be modelled by BSIM well and consequently BSIM-predicted SRTs may come out poorer in the ${\text{S}}_{0}{\text{N}}_{\text{20TB}}$ scenario. Another possible explanation is the usage of the Hagerman and Olofsson (2004) method to process speech and noise through the algorithms, in particular the (nonlinear) hearing aid compressor in the MHA, in order to provide separate speech and noise signals to BSIM. A requirement to keep the error of this method low is that the system under investigation should be as linear as possible. Although Hagerman and Olofsson (2004) also used a compressive hearing aid in their study, which they termed as ”quasi linear”, such a compressor may have influence on the calculated SNR. For positive input SNR, the compressor reduces the SNR at the output (Hagerman and Olofsson, 2004), requiring the input SNR to increase to obtain same speech intelligibility. That may explain at least parts of the overestimation of SRTs in the ${\text{S}}_{0}{\text{N}}_{20TB}$ scenario, because predictions are largely positive there.

On the other hand, BSIM underestimated the benefit of using beamformer algorithms (r = 0.91, p<0.01, RMSE = 2.9 dB) consistently across all spatial scenarios. In the ${\text{S}}_{0}{\text{N}}_{+90}$ scenario, BSIM predicted poorer SRTs for using the MVDR compared to the ADM. This was caused by a higher SNR-improvement of using the MVDR on the CI side compared to HA side. This resulted in higher SNRs on the CI side compared to the HA side, which resulted in the SII-reference weighting function determining that the listener would focus on their CI side rather than the HA side, increasing calculated electroacoustic SII reference needed by BSIM to achieve 50% speech recognition, and the predicted SRT as a result. Such issues were not encountered by using FADE.

### 4.3 Possible model applications

The model approaches proposed in the present study may have several applications, including, e.g. its applications in clinics, fundamental research and algorithm development.

For clinics, testing time of different beamformer settings and algorithms might be reduced, by using model predictions for the different beamformer settings and testing on the largest predicted differences for personalized algorithm settings. Similarly, for CI candidates with usable acoustic hearing contralaterally, preoperative predictions of the benefit of cochlear implantation for their speech understanding in realistic every-day life scenarios may help professionals and the patient about the decision for or against implantation. Postoperatively, models may guide which beamformer algorithm best to use in which situation, without extensive testing with the patient.

For fundamental research, the model comparison offers insights into which aspects of spatial speech intelligibility can be explained by better-ear-listening, which ones give indication for binaural integration, and which ones require binaural fine-structure processing. Such evaluations can not always be straight-forwardly inferred from just measured data, especially in complex acoustic scenarios and in conjunction with preprocessing algorithms (Schädler et al., 2018).

For algorithm developers, good models that capture the functional aspects of binaural speech intelligibility within the patient might be used by algorithm developers to optimize or test their algorithms before patient-testing. This may include developers who try to improve aspects of binaural processing with beamformers, or even developers who enable or foster the patient’s own binaural fine-structure processing, for example by trying to enable sensitivity to interaural time differences (Angermeier, Hemmert, Zirn, 2021, Williges, Jürgens, Hu, Dietz, 2018).

## 5 Conclusions

The two model approaches FADE and BSIM were compared with the aim to predict absolute SRTs and SRT-benefits due to beamformers in spatial scenarios for bimodal CI users. Experimental data of Zedan et al. (2021) was used as a comparison. The following conclusions can be drawn:

• FADE was able to predict absolute SRTs with very high accuracy ab-initio. This means that no further calibration needed to be done to the model. A simple concatenation of extracted features from electrically and acoustically stimulated side was sufficient.
• BSIM showed overall good, but somewhat lower accuracy than FADE for predicting absolute SRTs. Largest deviations occured in the complex ${\text{S}}_{0}{\text{N}}_{\text{20TB}}$ condition, where BSIM predicted much poorer SRTs than measured.
• Both models provided accurate predictions of the SRT-benefit that bimodal CI users get from beamformers. These predicted SRT-benefits are matching the average iSNR-improvement. This means that if only the benefit (i.e., the improvement relative to the SRT without beamforming) should be predicted, the average iSNR may be a preferable measure, because it is a much simpler approach than FADE and BSIM.

## CRediT authorship contribution statement

**Ayham Zedan:** Data curation, Formal analysis, Investigation, Methodology, Software, Validation, Visualization, Writing – original draft. **Tim Jürgens:** Conceptualization, Funding acquisition, Methodology, Project administration, Supervision, Writing – review & editing. **Ben Williges:** Methodology, Resources, Software, Writing – review & editing. **David Hülsmeier:** Methodology, Resources, Software, Writing – review & editing. **Birger Kollmeier:** Conceptualization, Funding acquisition, Methodology, Project administration, Resources, Supervision, Writing – review & editing.

## References

[bib0001] Angermeier J., Hemmert W., Zirn S. (2021). Sound localization bias and error in bimodal listeners improve instantaneously when the device delay mismatch is reduced. Trends Hear.

[bib0002] ANSI (1997). ANSI S3.5–1997 methods for calculation of the speech intelligibility index. American National Standards Institute.

[bib0003] Balkenhol T., Wallhusser-Franke E., Rotter N., Servais J.J. (2020). Cochlear implant and hearing aid: objective measures of binaural benefit. Front Neurosci.

[bib0004] Baumgärtel R.M., Hu H., Krawczyk-Becker M., Marquardt D., Herzke T., Coleman G., Adiloglu K., Bomke K., Plotz K., Gerkmann T., Doclo S., Kollmeier B., Hohmann V., Dietz M. (2015). Comparing binaural pre-processing strategies II: speech intelligibility of bilateral cochlear implant users. Trends Hear.

[bib0005] Baumgärtel R.M., Krawczyk-Becker M., Marquardt D., Völker C., Hu H., Herzke T., Coleman G., Adiloglu K., Ernst S.M.A., Gerkmann T., Doclo S., Kollmeier B., Hohmann V., Dietz M. (2015). Comparing binaural pre-processing strategies I: instrumental evaluation. Trends Hear.

[bib0006] Beutelmann R., Brand T. (2006). Prediction of speech intelligibility in spatial noise and reverberation for normal-hearing and hearing-impaired listeners. J. Acoust. Soc. Am..

[bib0007] Beutelmann R., Brand T., Kollmeier B. (2009). Prediction of binaural speech intelligibility with frequency-dependent interaural phase differences. J. Acoust. Soc. Am..

[bib0008] Beutelmann R., Brand T., Kollmeier B. (2010). Revision, extension, and evaluation of a binaural speech intelligibility model. J. Acoust. Soc. Am..

[bib0009] Bitzer J., Simmer K., Kammeyer K.-D. (1999). IEEE International Conference on Acoustics, Speech, and Signal Processing.

[bib0010] Buechner A., Dyballa K.-H., Hehrmann P., Fredelake S., Lenarz T. (2014). Advanced beamformers for cochlear implant users: acute measurement of speech perception in challenging listening conditions. PLoS ONE.

[bib0011] Byrne D., Dillon H., Ching T., Katsch R., Keidser G. (2001). Nal-nl1 procedure for fitting nonlinear hearing aids: characteristics and comparisons with other procedures. J Am Acad Audiol.

[bib0012] Ching T.Y., Incerti P., Hill M., Hill M. (2004). Binaural benefits for adults who use hearing aids and cochlear implants in opposite ears. Ear Hear.

[bib0013] Ching T.Y., Incerti P., Hill M., van Wanrooy E. (2006). An overview of binaural advantages for children and adults who use binaural/bimodal hearing devices. Audiology and Neurotology.

[bib0014] Clark N.R., Brown G.J., Jürgens T., Meddis R. (2012). A frequency-selective feedback model of auditory efferent suppression and its implications for the recognition of speech in noise. J. Acoust. Soc. Am..

[bib0015] Denk F., Kollmeier B. (2021). The hearpiece database of individual transfer functions of an in-the-ear earpiece for hearing device research. Acta Acustica.

[bib0016] Devocht E.M., Janssen A.M., Chalupper J., Stokroos R.J., George E.L. (2016). Monaural beamforming in bimodal cochlear implant users: effect of (a)symmetric directivity and noise type. PLoS ONE.

[bib0017] Dieudonn B., Francart T. (2020). Speech understanding with bimodal stimulation is determined by monaural signal to noise ratios: no binaural cue processing involved. Ear Hear.

[bib0018] Doclo S., Kellermann W., Makino S., Nordholm S. (2015). Multichannel signal enhancement algorithms for assisted listening devices. IEEE Signal Process Mag.

[bib0019] Dunn C.C., Tyler R.S., Witt S.A. (2005). Benefit of wearing a hearing aid on the unimplanted ear in adult users of a cochlear implant. Journal of Speech, Language and Hearing Research.

[bib0020] Durlach N.I. (1963). Equalization and cancellation theory of binaural masking-level differences. J. Acoust. Soc. Am..

[bib0021] Elko G., Pong A.-T.N. (1995). Workshop on Applications of Signal Processing to Audio and Accoustics.

[bib0022] Ernst A., Anton K., Brendel M., Battmer R.-D. (2019). Benefit of directional microphones for unilateral, bilateral and bimodal cochlear implant users. Cochlear Implants Int.

[bib0023] Francart T., Brokx J., Wouters J. (2008). Sensitivity to interaural time differences with combined cochlear implant and acoustic stimulation. J. Assoc. Res. Otolaryngol..

[bib0024] Francart T., McDermott H.J. (2013). Psychophysics, fitting, and signal processing for combined hearing aid and cochlear implant stimulation. Ear Hear.

[bib0025] Fredelake S., Hohmann V. (2012). Factors affecting predicted speech intelligibility with cochlear implants in an auditory model for electrical stimulation. Hear. Res..

[bib0026] Gifford R.H., Dorman M., Sheffield S.W., Teece K., Olund A. (2014). Availability of binaural cues for bilateral implant recipients and bimodal listeners with and without preserved hearing in the implanted ear. Audiol Neurootol.

[bib0027] Greenberg J.E., Peterson P.M., Zurek P.M. (1993). Intelligibility-weighted measures of speech to interference ratio and speech system performance. J. Acoust. Soc. Am..

[bib0028] Grimm G., Herzke T., Berg D., Hohmann V. (2006). The master hearing aid: a PC-based platform for algorithm development and evaluation. Acta Acustica united with Acustica.

[bib0029] Hagerman B., Olofsson A. (2004). A method to measure the effect of noise reduction algorithms using simultaneous speech and noise. Acta Acustica united with Acustica.

[bib0030] Hauth C., Brand T. (2018). Modeling sluggishness in binaural unmasking of speech for maskers with time-varying interaural phase differences. Trends Hear.

[bib0031] Herzke T., Hohmann V. (2005). Effects of instantaneous multiband dynamic compression on speech intelligibility. Journal on Advances in Signal Processing.

[bib0032] van Hoesel R.J.M. (2012). Contrasting benefits from contralateral implants and hearing aids in cochlear implant users. Hear. Res..

[bib0033] Holube I., Kollmeier B. (1996). Speech intelligibility prediction inhearing-impaired listeners based on a psychoacoustically motivated perception model. J. Acoust. Soc. Am..

[bib0034] Hoppe U., Hocke T., Digeser F. (2018). Bimodal benefit for cochlear implant listeners with different grades of hearing loss in the opposite ear. Acta Otolaryngol..

[bib0035] Hülsmeier D., Buhl M., Wardenga N., Warzybok A., Schädler M.R., Kollmeier B. (2021). Inference of the distortion component of hearing impairment from speech recognition by predicting the effect of the attenuation component. Int J Audiol.

[bib0036] Hülsmeier D., Warzybok A., Kollmeier B., Schädler M.R. (2020). Simulations with fade of the effect of impaired hearing on speech recognition performance cast doubt on the role of spectral resolution. Hear. Res..

[bib0037] Jeffress L.A. (1948). A place theory of sound localization. J Comp Physiol Psychol.

[bib0038] Jürgens T., Brand T. (2009). Microscopic prediction of speech recognition for listeners with normal hearing in noise using an auditory model. J. Acoust. Soc. Am..

[bib0039] Jürgens T., Hohmann V., Bchner A., Nogueira W. (2018). The effects of electrical field spatial spread and some cognitive factors on speech-in-noise performance of individual cochlear implant users-a computer model study. PlosOne.

[bib0040] Jürgens T., Wesarg T., Oetting D., Jung L., Williges B. (2021). Spatial speech-in-noise performance in simulated single-sided deaf and bimodal cochlear implant users in comparison with real patients. Int J Audiol.

[bib0041] Kayser H., Ewert S.D., Anemller J., Rohdenburg T., Hohmann V., Kollmeier B. (2009). Database of multichannel in-ear and behind-the-ear head-related and binaural room impulse responses. EURASIP J Adv Signal Process.

[bib0042] Kock W.E. (1950). Binaural localization and masking. J. Acoust. Soc. Am..

[bib0043] Kollmeier B., Kiessling J. (2018). Functionality of hearing aids: state-of-the-art and future model-based solutions. Int J Audiol.

[bib0044] Kollmeier B., Schädler M.R., Warzybok A., Meyer B.T., Brand T. (2016). Sentence recognition prediction for hearing-impaired listeners in stationary and fluctuation noise with fade: empowering the attenuation and distortion concept by plomp with a quantitative processing model. Trends Hear.

[bib0045] Laback B., Egger K., Majdak P. (2015). Perception and coding of interaural time differences with bilateral cochlear implants. Hear. Res..

[bib0046] Leigh J.R., Moran M., Hollow R., Dowell R.C. (2016). Evidence-based guidelines for recommending cochlear implantation for postlingually deafened adults. Int J Audiol.

[bib0047] Meddis R. (2006). Auditory-nerve first-spike latency and auditory absolute threshold: a computer model. J. Acoust. Soc. Am..

[bib0048] Moore B., Alcntara J., Stone M., Glasberg B. (1999). Use of a loudness model for hearing aid fitting: II. hearing aids with multi-channel compression. Br J Audiol.

[bib0049] Nogueira W., Bchner A., Edler B. (2005). Audio Engineering Society.

[bib0050] Schädler M., Warzybok A., Hochmuth S., B K. (2015). Matrix sentence intelligibility prediction using an automatic speech recognition system. Int. J. Audiol..

[bib0051] Schädler M.R. (2015). Robust automatic speech recognition and modeling of auditory discrimination experiments with auditory spectro-temporal features.

[bib0052] Schädler M.R., Hülsmeier D., Warzybok A., Kollmeier B. (2020). Individual aided speech-recognition performance and predictions of benefit for listeners with impaired hearing employing fade. Trends Hear.

[bib0053] Schädler M.R., Hülsmeyer D., Warzybok A., Hochmuth S., Kollmeier B. (2016). Microscopic multilingual matrix test predictions using an asr-based speech recognition model. Proceedings of Interspeech.

[bib0054] Schädler M.R., T W.A., Kollmeier B. (2018). Objective prediction of hearing aid benefit across listener groups using machine learning: speech recognition performance with binaural noise-reduction algorithms. J. Acoust. Soc. Am..

[bib0055] Schädler M.R., Warzybok A., Ewert S.D., Kollmeier B. (2016). A simulation framework for auditory discrimination experiments: revealing the importance of across-frequency processing in speech perception. J. Acoust. Soc. Am..

[bib0056] Vroegop J.L., Homans N.C., Goedegebure A., Dingemanse J.G., Immerzeel T.v., van der Schroeff M.P. (2018). The effect of binaural beamforming technology on speech intelligibility in bimodal cochlear implant recipients. Audiology and Neurotology.

[bib0057] Völker C., Warzybok A., Ernst S.M.A. (2015). Comparing binaural pre-processing strategies III: speech intelligibility of normal-hearing and hearing-impaired listeners. Trends Hear.

[bib0058] Wagener K., Brand T., Kollmeier B. (1999). Entwicklung und evaluation eines satztests for die deutsche sprache II: optimierung des oldenburger satztests [Development and Evaluation of a Sentence Test for the German Language II: Optimization of the Oldenburg Sentence Test]. Zeitschrift for Audiologie/Audiological Acoustics.

[bib0059] Weissgerber T., Rader T., Baumann U. (2017). Effectiveness of directional microphones in bilateral/bimodal cochlear implant users-impact of spatial and temporal noise characteristics. Otology and Neurotology.

[bib0060] Williges B., Dietz M., Hohmann V., Jürgens T. (2015). Spatial release from masking in simulated cochlear implant users with and without access to low-frequency acoustic hearing. Trends Hear.

[bib0061] Williges B., Jürgens T., Hu H., Dietz M. (2018). Coherent coding of enhanced interaural cues improves sound localization in noise with bilateral cochlear implants. Trends Hear.

[bib0062] Williges B., Wesarg T., Jung L., Geven L.I., Radeloff A., Jürgens T. (2019). Spatial speech-in-noise performance in bimodal and single-sided deaf cochlear implant users. Trends Hear.

[bib0063] Yoon Y.-S., Shin Y.-R., Gho J.-S., Fu Q.-J. (2015). Bimodal benefit depends on the performance difference between a cochlear implant and a hearing aid. Cochlear Implants Int.

[bib0064] Zaleski-King A., Goupell M.J., Barac-Cikoja D., Bakke M. (2019). Bimodal cochlear implant listeners’ ability to perceive minimal audible angle differences. J Am Acad Audiol.

[bib0065] Zamaninezhad L., Hohmann V., Bchner A., Schädler M.R., Jürgens T. (2017). A physiologically-inspired model reproducing the speech intelligibility benefit in cochlear implant listeners with residual acoustic hearing. Hear. Res..

[bib0066] Zedan A., Jürgens T., Williges B., Kollmeier B., Wiebe K., Galindo J., Wesarg T. (2021). Speech intelligibility and spatial release from masking improvements using spatial noise reduction algorithms in bimodal cochlear implant users. Trends Hear.

[bib0067] Zedan A., Williges B., Jürgens T. (2018). Modeling speech intelligibility of simulated bimodal and single-sided deaf cochlear implant users. Acta Acustica united with Acustica.

[bib0068] Zirn S., Angermeier J., Arndt S., Aschendorff A., Wesarg T. (2019). Reducing the device delay mismatch can improve sound localization in bimodal cochlear implant/hearing-aid users. Trends Hear.

